# Microscope-assisted percutaneous microchannel treatment of lumbar ligamentum flavum cyst: A case report

**DOI:** 10.1097/MD.0000000000045859

**Published:** 2025-11-07

**Authors:** Nana Guo, Yonghua Wang, Gang Gao, Na Liu, Yanhan Guo, Xiangfu Kong, Wupeng Yang, Yongjiang Wang, Tingxin Zhang

**Affiliations:** a Critical Care Medicine, Ordos Central Hospital, Ordos, China; b Department of Orthopedics, Ordos Central Hospital, Ordos, China; c Medical Image Center, Ordos Central Hospital, Ordos, China; d Anesthesiology Department, Ordos Central Hospital, Ordos, China.

**Keywords:** case report, ligamentum favum cyst, lumbar, percutaneous microchannel

## Abstract

**Rationale:**

The main treatment for lumbar ligamentum flavum cyst is surgery. The purpose of surgery is to remove the cyst and relieve nerve root compression. In recent years, microscope-assisted percutaneous microchannel therapy has become a minimally invasive and effective alternative to open surgery. However, the efficacy of this surgery for lumbar ligamentum flavum cysts has not yet been determined.

**Patient concerns:**

We report a case of a 61-year-old male patient who was admitted due to lower back pain, right lower limb pain, and numbness that had persisted for 6 months. Magnetic resonance imaging showed a ligament cyst compressing the nerve at the L4/5 intervertebral level.

**Diagnoses:**

Lumbar ligament cyst.

**Interventions:**

Cysts were resected percutaneously via microchannels with the aid of a microscope.

**Outcomes:**

The clinical effect was good after 2 year of follow-up. The patient’s clinical effect after surgery was significantly improved compared with that before surgery, and postoperative lumbar spine magnetic resonance imaging showed complete removal of the cyst.

**Lessons:**

Lumbar ligament cyst is a rare spinal disease with many case reports. At present, its pathogenesis is not clear, and the current consensus is that it is mostly related to spinal degenerative diseases. The main treatment for lumbar ligamentum flavum cyst is surgery. Microscope-assisted percutaneous microchannel surgical resection of lumbar ligamentum flavum cyst is a safe and effective method that can protect the normal anatomical structure of the lumbar spine to the maximum extent, without destroying the stability of the spine, while reducing surgical trauma.

## 1. Introduction

Spinal ligamentum flavum cyst is a rare degenerative spinal lesion found in the spinal canal.^[1]^ The exact cause of this condition is still unclear, but it is believed to be linked to degeneration of the ligamentum flavum.^[2,3]^ Professor Moile first reported on spinal ligamentum flavum cysts in 1976.^[4]^ Spinal ligamentum flavum cysts commonly occur in the lumbar spine, most commonly at the L4-5 and L5-S1 levels.^[5]^ While many lumbar ligamentum flavum cysts do not present with specific clinical symptoms, some may result in lumbosacral pain. When nerve root compression occurs, it can lead to typical symptoms of nerve root compression, resembling those of lumbar disc herniation and potentially causing misdiagnosis. Treatment for lumbar ligamentum flavum cysts typically involves surgical removal of the cyst to alleviate nerve compression. The technique of percutaneous microchannel surgical resection of cysts under microscope is based on X-ray positioning, using an expansion sleeve to bluntly separate the cyst through the muscle space step by step, and insert the microchannel along the expansion sleeve and fix it. The cyst is then exposed under a microscope and removed. However, the efficacy of this surgery for lumbar ligamentum flavum cysts has not yet been determined. In this case report, a patient with a ligamentum flavum cyst at the L4-5 level underwent microscopically assisted percutaneous microchannel resection in the lumbar spine. Following the operation, the patient experienced significant relief in symptoms and reported a satisfactory outcome.

## 2. Methods

The patient, a 61-year-old male, was admitted to the hospital due to lower back pain accompanied by radiating pain and numbness in the right lower limb for 6 months. The patient is a driver with no history of chronic diseases. After 3 months of rest, oral pain medication, and acupuncture treatment, the pain and numbness did not alleviate. The patient’s symptoms gradually worsened and he is currently limited in standing and walking, with no improvement after conservative treatment. Physical examination revealed tenderness and percussion pain in the L4-5 spinal region and limited lumbar extension. The skin sensation on the lateral calf and dorsum of the right lower limb was reduced. The tibialis anterior muscle strength of the right lower limb was grade IV, the extensor pollicis longus muscle strength of the right lower limb was grade III, the tibialis anterior muscle strength of the left lower limb was grade V, and the extensor pollicis longus muscle strength of the left lower limb was grade V. For our muscle strength testing, we utilized the Manual Muscle Testing scale. The right lower limb straight leg raising test at 40° was positive and the straight leg raising test of the right lower extremity was negative. Physiological reflexes of the lower limbs were elicited normally, but pathological reflexes were not elicited. Based on the Japanese Orthopaedic Association (JOA) scoring system, the neurological function score of the patient was 10 points. Back pain Visual Analogue Scale (VAS) score is 7 points, right leg pain VAS score is 8 points. Preoperative lumbar spine Oswestry Disability Index (ODI) score was 60%.

**Figure 1.**
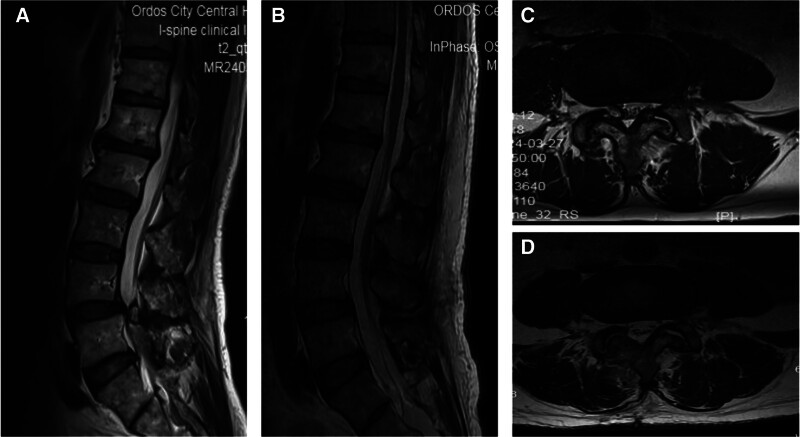
Preoperative and postoperative lumbar spine MRI. (A and C) Sagittal and axial T2-weighted imaging shows a round signal shadow on the right side of the spinal canal at the L4/5 level. (B and D) Three months after surgery, sagittal and axial T2-weighted MRI of the lumbar spine showed that the intraspinal mass at the L4/5 level was completely removed without recurrence. MRI = magnetic resonance imaging.

On January 10, 2023, magnetic resonance imaging (MRI) showed a ligament cyst compressing the nerve at the L4/5 intervertebral level (Fig. 1). A diagnosis of lumbar ligamentum flavum cysts was made. The surgical treatment plan was microscope-assisted percutaneous microchannel to remove the L4/5 ligamentum flavum cyst.

## 3. Surgical treatment

**Figure 2.**
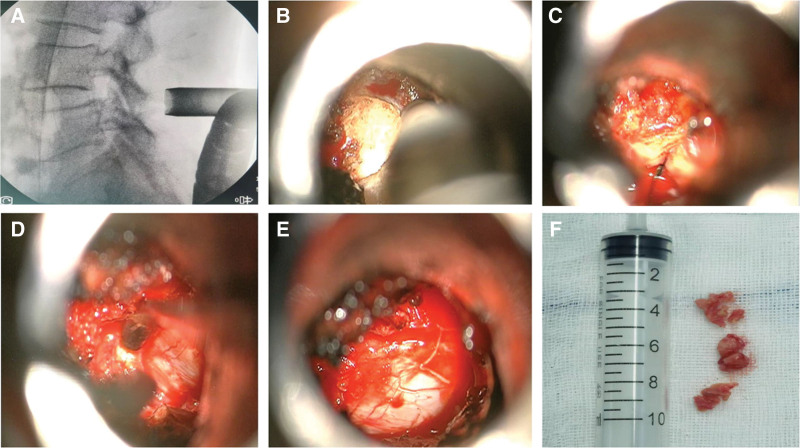
Intraoperative situation. (A) Lateral view shows channel localization at L4/5 level. (B) Removal of the lamina and spinous process bases using a powered system. Exposed ligamentum flavum. (C) Removal of ligamentum flavum using Kerrison rongeurs. (D) The round mass in the ligamentum favum was seen under the microscope, with obvious adherent to the nerve root and dural sac. (E) Nerve root relaxation after the mass was removed. (F) Dense fibrous connective tissue removed during surgery.

**Figure 3.**
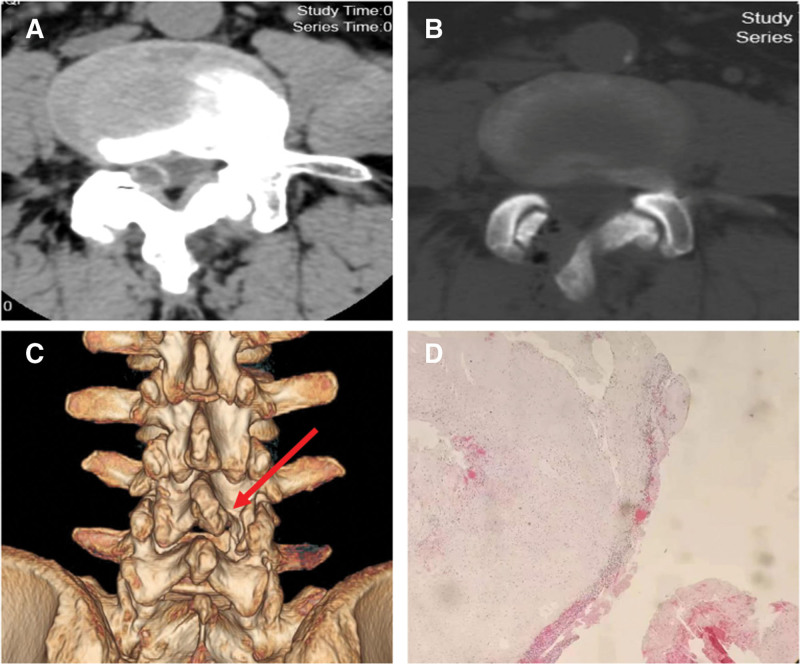
(A) Preoperative CT showed a round low-density shadow on the right side of the spinal canal at the L4/5 level. (B and C) Three months after the operation, lumbar spine CT and 3-dimensional reconstruction showed that the L4/5 level tumor was completely removed, with no recurrence, and the facet joints were well preserved. (D) The postoperative pathological examination results showed cystic wall-like changes in connective tissue without epithelial cell tissue. CT = computed tomography.

On January 16, 2023, the L4/5 ligament cyst was resected under microscope-assisted percutaneous microchannel after general anesthesia. After successful general anesthesia, the patient was placed in a prone position with the abdomen suspended, and a neurophysiological monitoring system was established. The C-arm accurately locates the spinal canal at the L4-5 level, and makes a longitudinal skin incision 2.5 cm right of the posterior midline with a length of about 1.8 cm. The subcutaneous and fascia were incised sequentially, and the paravertebral expansion cannula was used to bluntly separate the muscle layer step by step through a Gram needle, and a surgical microchannel (diameter of 1.6 cm) was inserted under the guidance of the expansion cannula (Fig. 2). The expansion sleeve was placed along the periphery and connected to the fixation rod through a serpentine chain. It was then fixed beside the bed. The C-arm was used to confirm that the channel was at the L4-5 level. Adjust the microscope, use a drill to remove the root of the 4 spinous processes of the lumbar vertebra and the lower edge of the L4 lamina, and use a drill to open the lamina based on the preoperative imaging data. The ligamentum flavum is then removed using an articulator. The ligamentous cyst is exposed, and then the ligamentous cyst is completely removed and the nerve root is decompressed (Fig. 2). At the end of the operation, the pain in the right lower limb was significantly relieved. The intraoperative blood loss was 15 ml. Pathological examination revealed fibrous tissue hyperplasia accompanied by vitreous degeneration and focal mucoid degeneration, confirming the specimen as a ligamentum flavum cyst (Fig. 3).

## 4. Results

The patient experienced immediate relief from pain in his lower limbs and back after surgery. At the last follow-up, the patient’s VAS score for low back pain and leg pain was 2 points. Two weeks after the operation, the muscle strength of the right lower limb improved to level IV±. Before surgery, the patient’s JOA score was 10 points. After 24 months of follow-up, the JOA score improved to 25 points, and the JOA score improvement rate after treatment reached 74%. The patient’s preoperative ODI score was 60%. After 24 months of follow-up, the ODI score improved significantly, reaching 10% (Table 1). Postoperative computed tomography and MRI showed that the L4-5 segment ligamentum flavum cyst was completely removed without recurrence, the facet joints were well preserved, and the stability of the lumbar spine was not damaged (Figs. 1 and 3).

**Table 1:** Patient follow-up results.

	Preoperative	Postoperative				
		1 wk	1 mo	3 mo	12 mo	24 mo
Back pain VAS	8	4	3	2	2	2
Leg pain VAS	8	4	3	2	2	2
ODI	60	29	16	13	10	9
JOA	10	16	20	21	24	25

JOA = Japanese Orthopaedic Association (0–29), ODI = Oswestry Disability Index (0–100%), VAS = Visual Analog Score (0–10).

## 5. Discussion

Ligamentum flavum cyst is a type of facet joint cyst. Ligamentum flavum cyst may be caused by excessive mobility of the articular surface and myxomatous degeneration of the ligamentum flavum.^[6,7]^ Lumbar ligamentum flavum cyst is relatively rare in clinical practice. It commonly occurs in middle-aged and elderly people.^[8]^ It can occur in the cervical, thoracic and lumbar vertebrae, but it is most common in the lower lumbar vertebrae, and the L4/5 segment is the most common, because the L4/5 segment has the greatest range of motion among all lumbar vertebrae.^[9,10]^ Although the pathogenesis of lumbar ligamentum flavum cyst is still unclear, some studies believe that osteoarthritis, spinal degenerative disease, spondylolisthesis and degenerative disc disease have a certain correlation in the formation of spine-related cystic disease.^[11–13]^ Due to the influence of factors such as type II collagen deposition and loss of elastic fibers in the ligamentum flavum, spinal stability is further affected, leading to the formation of cysts and transforming into more severe degeneration.^[14]^ Lumbar ligamentum flavum cysts have no specific clinical manifestations. The clinical manifestations mainly depend on the location, size and growth rate of the cyst, and most of them have a slow onset.^[15]^ Most cases are found by MRI examination, and a few cases may also have acute onset due to intracystic bleeding.^[16]^

The radiological diagnosis of ligamentum flavum cyst is relatively difficult. Ordinary plain X-ray films mostly show signs of spinal degeneration. Myelography showed intraspinal epidural nerve root compression and lacked the specific findings of a ligamentum flavum cyst. MRI plays an important role in diagnosing ligamentum flavum cysts.^[8]^ Ligamentum flavum cysts show high signal on T2WI images and low signal or isointense changes on T1WI images. Mild enhancement of the tumor can be seen on enhancement, which is often accompanied by spinal degeneration.^[9]^ Conventional MRI plain scans are often at the intervertebral disc level, so thin-section scans are not performed on the ligamentum flavum level, so there is a possibility of missed diagnosis in the diagnosis of ligamentum flavum cyst. Lumbar ligamentum flavum cyst is prone to misdiagnosis due to its clinical rarity and imaging characteristics.^[17]^ A careful physical examination should be performed to identify common diseases such as lumbar disc herniation, lumbar spinal stenosis, and postoperative adjacent spondylosis. It also needs to be differentiated from lumbar facet joint synovial cysts, ganglion cysts, and intraspinal tumors. Synovial cysts and ganglion cysts both originate from facet joints, but they are pathologically different; ligamentum flavum cysts originate from the ligamentum flavum, and most cysts are located in the ligamentum flavum, which are relatively independent and have no connection with the facet joints.

In terms of treatment, conservative treatment is feasible when there are no symptoms or mild symptoms. If there are typical clinical symptoms and a clear diagnosis, surgical resection is the first choice. The ultimate goal of surgery is to completely decompress the compressed nerve root. If completely removed, the probability of recurrence of the ligamentum flavum cyst is still relatively low. Because lumbar ligamentum flavum cyst is clinically rare, there is currently no clear standard for its surgical method. Reviewing the previous literature (Table 2), surgical approaches include posterior laminectomy decompression, microendoscopic discectomy, and transforaminal lumbar interbody fusion.^[14,18–26]^ Lumbar interbody fusion and endoscopic spinal cyst resection via interlaminar approach. In addition, in view of the correlation between ligamentum flavum cyst and spinal instability, it has been controversial whether it is necessary to perform spinal internal fixation at the same time as resection of ligamentum flavum cyst during surgery. Regardless of whether it is internally fixed or not, open surgery cannot avoid major trauma. Although the cyst can be removed relatively completely, scar adhesion is often difficult to avoid in the later stage, and even symptoms may remain due to scarring, resulting in increased surgical costs, intraoperative bleeding, and increased recovery time. In percutaneous microchannel surgery, an expansion sleeve is generally used to expand step by step along the muscle gap. Since the muscle tissue is separated by step-by-step expansion, the normal arrangement of muscle fibers is not disrupted. And the paravertebral muscles will not be peeled off from the spinous process and lamina in a large area, and the attachment points and integrity of the muscles are retained. The degree of muscle damage is significantly reduced, the incidence of postoperative paravertebral muscle atrophy is significantly reduced, the physiological function of paravertebral soft tissue is effectively preserved, and the dynamic function of muscle tissue is almost unaffected.^[27]^ Only a small bone window is opened in the vertebral plate, which keeps the structural stability of the spine intact, avoids potential complications such as poor healing, cerebrospinal fluid leakage, and infection in the surgical area, and further shortens the length of hospital stay and the time for getting out of bed.

**Table 2:** Reported 19 cases of ligament flavum cysts occurring in the lumbar spine.

Literature	No. of cases	Surgical method	Level and cases
Baker and Hanson^[18]^	1	PLD	L5-S1(1)
Vernet et al^[19]^	6	PLD	L3-4(1), L4-5(4), L5-S1(1)
DiMaio et al^[14]^	4	PLD	L3-4(2), L5-S1(2)
Taha et al^[1]^	1	PLD	L3-4(1)
Seo et al^[16]^	1	PLD	L5-S1(1)
Shah et al^[9]^	1	MED	L4-5(1)
Kalidindi et al^[20]^	3	TLIF	L3-4(1), L4-5(2)
Yang et al^[21]^	1	TLIF	L4-5(1)
Singh et al^[22]^	1	PLD	L4-5(1)

MED = microendoscopic discectomy, PLD = surgical approaches included posterior laminectomy decompression, TLIF = transforaminal lumbar interbody fusion.

We believe that percutaneous microchannel minimally invasive surgical resection of ligamentum flavum cyst has the following characteristics: The channel is inserted through the muscle space through a skin incision of <2.0 cm, and the direction of the channel can be adjusted to obtain a multi-angle operating space. However, it is necessary to position accurately before surgery, plan the puncture path in advance, and puncture the target point to avoid puncturing the cyst wall. The operating channel is a closed whole, which embeds soft tissue around the tissue while compressing the surrounding tissue to reduce bleeding. Only a small bone window is opened on the vertebral plate, which does not destroy the stable structure of the spine. Due to the limitations of channels and instruments, it is difficult to completely remove the cyst, and the cyst wall may need to be destroyed. After the contents have drained out, the cyst wall should be removed and sent for pathological examination.

## 6. Conclusion

In summary, percutaneous microchannel surgical resection of lumbar ligamentum flavum cyst under a microscope is a safe and effective treatment method. During the operation, only the paravertebral structures on one side need to be bluntly dissected, the lamina on one side is exposed, and part of the bony structure of the lamina is removed. It does not damage the ligaments and surrounding attachment structures, so it can protect the normal anatomical structures to the greatest extent, not destroy the stability of the spine, and reduce surgical trauma. It provides clinicians with a new minimally invasive option for the treatment of lumbar ligamentum flavum cyst.

## Author contributions

**Data curation:** Nana Guo, Yonghua Wang, Gang Gao, Yanhan Guo, Xiangfu Kong, Wupeng Yang, Yongjiang Wang, Tingxin Zhang.

**Formal analysis:** Nana Guo, Yonghua Wang, Na Liu, Yanhan Guo, Xiangfu Kong, Wupeng Yang.

**Methodology:** Yongjiang Wang, Tingxin Zhang.

**Software:** Gang Gao, Na Liu, Xiangfu Kong, Wupeng Yang, Yongjiang Wang.

**Writing – original draft:** Nana Guo.

**Writing – review & editing:** Yonghua Wang, Tingxin Zhang.
